# Metalplant Farms Chemicals for the Clean Energy Transition

**DOI:** 10.1021/acscentsci.5c00049

**Published:** 2025-01-28

**Authors:** Diana Kruzman

When Sahit Muja was a child growing up in the Tropojë region of northern Albania, Lulja e Qenit was no more than a weed. The bushy green plant with tiny yellow blossoms grew along the side of the road and invaded farm fields and pastures; animals refused to eat it, and locals often tried to burn it.

In 2021, Muja, a businessman and developer, got a call from US climate tech entrepreneur Eric Matzner. Years earlier, one of Matzner’s start-ups had been interested in sourcing a carbon-trapping mineral called olivine from a quarry Muja owned in Tropojë.

That start-up ended up sourcing olivine from a different location, and their partnership never blossomed, but Matzner now thought Muja could help him with a different problem: the olivine the start-up was using to capture carbon along coastlines was releasing nickel into the ocean, and scientists feared that could affect corals offshore. But, as Matzner excitedly told Muja over the phone, Albania’s Lulja e Qenit could be the solution. The plant, also known by the species name *Odontarrhena chalcidica*, is a nickel hyperaccumulator, meaning that it can draw up the metal from the soil and deposit it in its leaves and stems at high concentrations.

**Figure:**
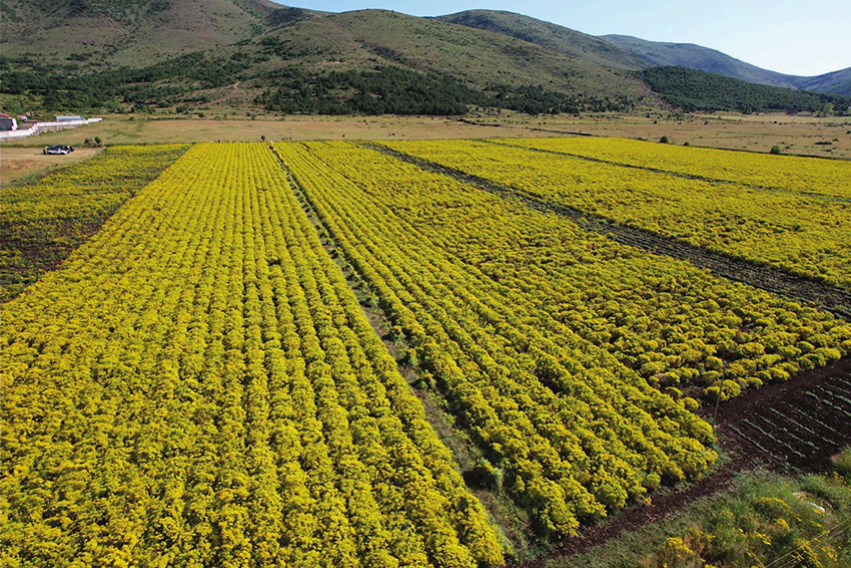
*Odontarrhena chalcidica* blooms in Metalplant’s fields in the spring. Credit: Sahit Muja.

Eventually, the two realized that using plants to collect nickel could be a business of its own. That same year, Muja and Matzner, along with operations executive Laura Wasserson, founded Metalplant, a start-up that specializes in a technique called phytomining—a method of extracting metals from the soil using plants—which they apply to farming fields of *O. chalcidica* and harvesting the valuable nickel contained within it. Albania’s nickel-rich soil makes it almost useless for growing food crops, but Muja and Matzner believe it is perfect for phytomining.

Traditional metal mining “goes against the thermodynamic gradient,” Matzner says. It uses chemicals and heat to extract metals from ores that naturally want to hold on to them. Phytomining, on the other hand, allows plants to do most of the work of extraction, reducing the energy input required.

Entrepreneurs are quickly commercializing this technique as demand for nickel grows for applications like electric vehicle batteries. In August, the US Advanced Research Projects Agency - Energy, or ARPA-E, granted $9.9 million to seven projects aimed at improving and scaling up phytomining on US soil. Metalplant was among them, with a proposal to increase *O. chalcidica's* yield and to genetically modify the plant to avoid the possibility of its becoming invasive once it takes root in the US.

**Figure:**
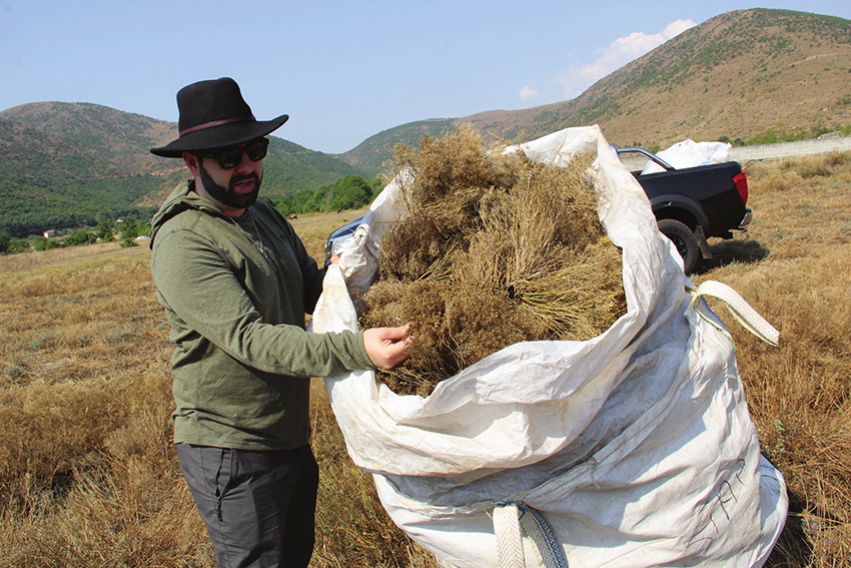
Eric Matzner harvesting *Odontarrhena chalcidica* in Tropojë, Albania. Credit: Diana Kruzman.

The company is growing at a time when the US seeks to boost the domestic supply chain for nickel and reduce imports while satisfying demand for the clean energy transition. The company appeals to a niche market, offering less carbon-intensive nickel for more conscious consumers. If it can build that market and scale up, the Metalplant team could lay the blueprint for the expansion of cleaner, more environmentally friendly nickel.

More than 700 nickel hyperaccumulators are known to science, but Metalplant chose *O. chalcidica* because it’s one of the best. The plant’s biomass can contain up to 2% nickel by dry weight, and its high yield and quick growth can deliver between 200 and 400 kg of nickel per hectare in one growing season, according to Metalplant’s estimate.

The US Department of Agriculture’s Agricultural Research Service first developed the field of phytomining in the 1980s, but the effort was a victim of its own success. The agency halted an early project after *Alyssum*, a relative of *Odontarrhena* also sourced from Albania, flourished to the point of becoming an invasive species around an Oregon test site.

Now, Metalplant is reviving this technique, hoping to take advantage of the increasing demand for nickel as a critical element in electric vehicle batteries. That sets it apart from other phytomining ventures, such as the France-based start-up Econick, which mainly targets steelmakers. Metalplant plans to market its nickel as an alternative to that supplied by Indonesia, the biggest producer of the metal, whose mining operations are plagued by environmental and human rights abuses.

**Figure:**
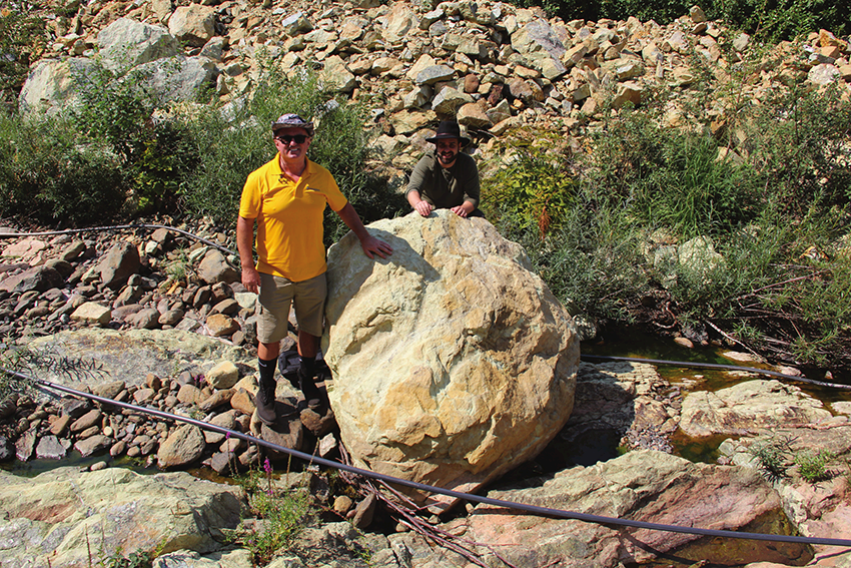
Sahit Muja (left) and Eric Matzner in Metalplant’s olivine quarry, about 40 min away from its fields. Credit: Diana Kruzman.

But before nickel can be sold, it needs to be extracted from the plant material. Phytomining companies, including Metalplant, generally start by burning the plant biomass to create a fine ash. Though Metalplant would not provide details of the exact method it uses beyond that step, researchers have successfully extracted nickel by either smelting the ash or dissolving it in sulfuric acid. In the acid method, nickel can be separated from the resulting leachate using a chelating resin that binds to the cations.

Regardless of the method used, the resulting product comes in a form that steel or battery makers can use immediately, such as a nickel salt or a high-purity nickel ingot, both of which Metalplant offers to buyers.

Burning the biomass releases greenhouse gases and somewhat tempers the climate benefits of the resulting metal. Metalplant counteracts this drawback with a carbon capture technique called enhanced rock weathering—the very process Matzner used in his previous start-up.

In the natural rock weathering process, rocks like olivine are weathered by rain and release minerals like calcium and magnesium. These minerals react with the carbon dioxide the rainwater has picked up from the air and produce compounds such as calcium carbonate. This alkaline substance is swept away by rivers and deposited in the ocean, where it is sequestered for up to 10,000 years.

To pull off enhanced rock weathering, Metalplant grinds up olivine sourced from a quarry about 40 min from its fields and spreads it on the soil where the nickel-loving plants grow. The olivine powder weathers naturally and releases nickel back into the soil, replenishing the soil supply for plants to take up through hyperaccumulation. Overall, for every kilogram of nickel Metalplant produces, it removes 200 kg of carbon dioxide from the atmosphere—creating a product the company calls NegativeNickel.

But challenges stand in the way of large-scale applications. For the time being, the cost of phytomining nickel far outweighs traditional mining due to its smaller production capacity. Rather than competing directly with major producers in Indonesia, Matzner says that Metalplant is focused on getting commitments from companies such as electric vehicle battery makers or green steel manufacturers that are willing to pay a “green premium” for carbon-negative nickel.

At the same time, he hopes future efforts to combat climate change, such as putting a price on carbon, could make conventionally mined nickel not worth the cost. “My hope would be that if they had to pay for the carbon footprint of their nickel, if they had to pay the cost of the deforestation of their nickel, their nickel would not be competitive,” Matzner says.

**Figure:**
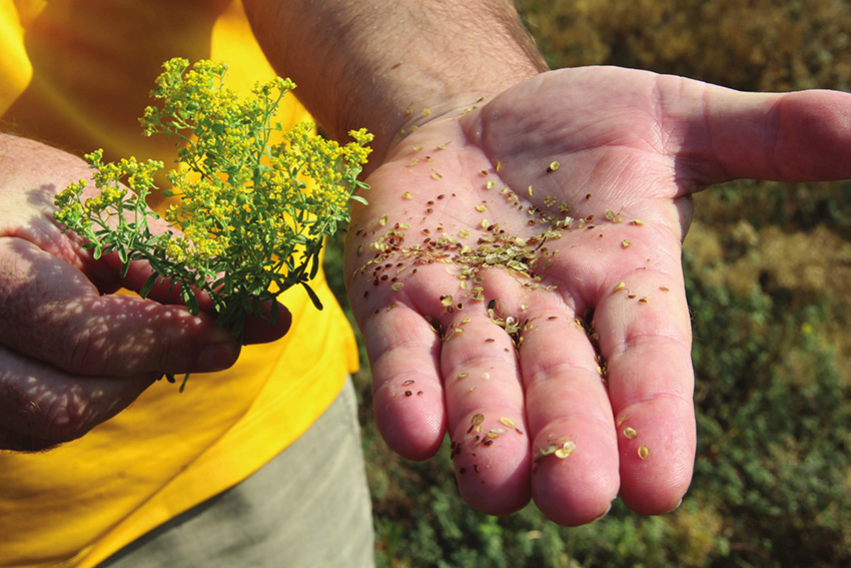
Metalplant collects *Odontarrhena chalcidica* seeds from its fields and stores them for future crops. Credit: Diana Kruzman.

Colleen Doherty, a plant biochemist at North Carolina State University whose lab is using plants to mine rare earth elements, believes commercializing phytomining will involve more than just finding willing buyers or perfecting the science. “There’s this perception of plant mining as hippie dippy—that it’s not gonna work, it’s not realistic,” Doherty says. “And phytomining still has to contend with that.”

Despite these barriers, Muja sees phytomining as an opportunity not only to secure minerals for the energy transition but also to provide opportunities for people living in areas like northern Albania, where the soil makes agriculture difficult and unprofitable.

“As an Albanian myself, I try to create value to my land and my people instead of extracting those resources for very little money,” Muja told C&EN on a ride through the Albanian countryside in early September.

He showed off Metalplant’s fields—with the harvest over, workers were concentrating on collecting *O. chalcidica* seeds. With the marriage of nickel-rich soils and nickel-loving plants, Muja says, “the future is really bright here.”

*Diana Kruzman is a freelance contributor to*Chemical & Engineering News, *an independent news publication of the American Chemical Society.*

